# Editorial: Interplays and Functions of Gaseous Mediators: From Underlying Mechanisms to Therapeutic Approaches in Cardiovascular Diseases

**DOI:** 10.3389/fphar.2022.925561

**Published:** 2022-05-23

**Authors:** Vincenzo Brancaleone, Emma Mitidieri, Andreas Papapetropoulos

**Affiliations:** ^1^ University of Basilicata, Potenza, Italy; ^2^ University of Naples Federico II, Naples, Italy; ^3^ National and Kapodistrian University of Athens, Athens, Greece

**Keywords:** hydrogen sulfide, nitric oxide, hypertension, heart failure, endothelium

The gaseous mediators nitric oxide (NO), hydrogen sulfide (H_2_S) and carbon monoxide (CO) constitute a family of molecules with unique features. Their production is endogenously regulated ensuring tight control of organ and tissue function. NO, CO and H_2_S have been shown to play important role in cardiovascular homoeostasis. Recent findings have highlighted the interaction between these gaseous molecules in modulating different signaling pathways. Gas poisoning resulting from acute CO build-up is associated with increases in reactive oxygen species (ROS) and reactive nitrogen species (RNS) evoking oxidative stress. These events target ferroproteins (FP) in endothelial, as well as in smooth muscle cells. FPs involved in gasotransmitter actions include ion channels and enzymes. In particular, impaired metabolism-blood flow coupling has been associated with CO-mediated modulation of BK_Ca_, K_ATP_, Kv_1.5_, and L-type Ca^2+^ channels (reviewed in Coburn). In addition, CO regulates enzyme activity; CO binds to catalase and peroxidases, inhibiting their ability to detoxify ROS, also affecting the levels of peroxynitrite and sulfide-related reactive molecules (reviewed in Coburn). The interaction between CO and H_2_S could also result from binding of CO to FPs like cystathionine-β-synthase (CBS), inhibiting their enzymatic activity. Indeed, this will reduce the biosynthesis of CBS-derived H_2_S, leading to impaired vascular function (reviewed in Coburn).

The inhibition of enzymatic activity is not the only effect associated with CO poisoning. In fact, chronic exposure to low levels of CO is associated with an increased risk of cardiac arrhythmia. In particular, CO generated by CO-releasing molecules (CORMs) prolongs the length of the potential of action in rat ventricular myocytes and induces early after-depolarization in ventricular myocytes from guinea pig (Al-Owais et al.). Interestingly, this effect has also been observed in human induced pluripotent stem cell derived cardiomyocytes and in computational tissue models (Al-Owais et al.). Thus, the effects of CORMs seem to facilitate the onset of arrhythmias, especially during heart failure.

Ischemia-reperfusion injury is one of the main causes of cardiac disease following myocardial infarction; recanalization of large vessels in thromboembolism is crucial to prevent extended damages. In this setting, a new delivery system as NO-loaded microbubbles is particularly efficient in improving the circulation time of NO *in vivo*. In particular, in this model, a significant decrease in the thrombus area and an increase in the recanalization rates and blood flow velocities have been observed (Liang et al.). Interestingly, this event is based on activation of endothelial nitric oxide synthase (eNOS). Of course, this effect could be therapeutically useful in diverse pathologies, including hypertension. In this case, therapy relies on renin-angiotensin system inhibitors and other antihypertensive drugs that unfortunately do not protect patients from developing heart failure. It is noteworthy, that in the study by Nguyen and coworkers, the treatment with sodium thiosulfate (STS) improved both hypertension and systolic function in a rat model of NO deficiency (Nguyen et al.).

In conclusion, in this issue further evidence relating to the role of NO, H_2_S and CO in the cardiovascular system is presented, clarifying some of the mechanisms that mediate their functions. In addition, an interplay between two or all three gaseous mediators taking place in the context of pathophysiological alterations of the cardiovascular system is also reported; further studies are needed to better exploit possible novel therapeutic interventions that could be clinically useful.

